# A reduction in Npas4 expression results in delayed neural differentiation of mouse embryonic stem cells

**DOI:** 10.1186/scrt453

**Published:** 2014-05-08

**Authors:** Thomas S Klaric, Paul Q Thomas, Mirella Dottori, Wai Khay Leong, Simon A Koblar, Martin D Lewis

**Affiliations:** 1 School of Molecular and Biomedical Science, The University of Adelaide, Adelaide, SA 5005, Australia; 2 Stroke Research Program, The University of Adelaide, Adelaide, SA 5005, Australia; 3 Centre for Neural Engineering, The University of Melbourne, Melbourne, VIC 3010, Australia

## Abstract

**Introduction:**

Npas4 is a calcium-dependent transcription factor expressed within neurons of the brain where it regulates the expression of several genes that are important for neuronal survival and synaptic plasticity. It is known that in the adult brain Npas4 plays an important role in several key aspects of neurobiology including inhibitory synapse formation, neuroprotection and memory, yet very little is known about the role of Npas4 during neurodevelopment. The aim of this study was to examine the expression and function of Npas4 during nervous system development by using a combination of *in vivo* experiments in the developing mouse embryo and neural differentiation of embryonic stem cells (ESCs) as an *in vitro* model of the early stages of embryogenesis.

**Methods:**

Two different neural differentiation paradigms were used to investigate Npas4 expression during neurodevelopment *in vitro*; adherent monolayer differentiation of mouse ESCs in N2B27 medium and Noggin-induced differentiation of human ESCs. This work was complemented by direct analysis of Npas4 expression in the mouse embryo. The function of Npas4 in the context of neurodevelopment was investigated using loss-of-function experiments *in vitro*. We created several mouse ESC lines in which Npas4 expression was reduced during neural differentiation through RNA interference and we then analyzed the ability of these Npas4 knockdown mouse ESCs lines to undergo neural differentiation.

**Results:**

We found that while Npas4 is not expressed in undifferentiated ESCs, it becomes transiently up-regulated during neural differentiation of both mouse and human ESCs at a stage of differentiation that is characterized by proliferation of neural progenitor cells. This was corroborated by analysis of Npas4 expression in the mouse embryo where the Npas4 transcript was detected specifically in the developing forebrain beginning at embryonic day 9.5. Finally, knockdown of Npas4 expression in mouse ESCs undergoing neural differentiation affected their ability to differentiate appropriately, resulting in delayed neural differentiation.

**Conclusions:**

Here we provide the first evidence that Npas4 is expressed during embryonic development and that it may have a developmental role that is unrelated to its function in the adult brain.

## Introduction

Neuronal PAS domain protein 4 (Npas4) is a transcription factor belonging to the basic helix-loop-helix Per-Arnt-Sim (bHLH PAS) family of regulatory proteins [1-3]. Members of this family are involved in a wide range of biological processes including embryonic development, cellular response to environmental stresses and circadian rhythm [4]. In the adult organism, expression of Npas4 is largely restricted to the brain [2,5,6] where it is expressed predominantly in excitatory neurons in response to neuronal activity-mediated calcium (Ca^2+^) signaling [7]. Unlike most other bHLH PAS proteins, which are continually expressed but are regulated at the post-translational level [4], Npas4 expression is regulated primarily at the level of transcription. Up-regulation of *Npas4* mRNA is independent of *de novo* protein synthesis, occurs within minutes and is dependent on nuclear Ca^2+^ signaling [8] making it an immediate-early gene that acts as a direct link between changes in intracellular Ca^2+^ levels and rapid changes in gene expression.

Through its regulation of various activity-dependent transcriptional programs, Npas4 is involved in several aspects of neuronal homeostasis. A number of studies have established the importance of Npas4 in activity-dependent neuroprotection [5,9,10], but there is now mounting evidence that Npas4 also plays a critical role in modulating synaptic plasticity. Npas4 was first implicated in synaptic plasticity when it was identified as a master regulator of inhibitory synapse formation [7]. More recently, it has been shown that Npas4 is induced by learning and also has a functional role in memory formation [8,11].

To date, there are few Npas4 target genes that have been experimentally validated, although several of the genes identified thus far have been implicated in neuronal plasticity. One component of the transcriptional program regulated by Npas4 is brain-derived neurotrophic factor (Bdnf) [7,12], a multifaceted neurotrophin that is important in the development and function of the nervous system due to its roles in neuronal survival, differentiation and synaptic plasticity [13-16]. Another is developmentally regulated brain protein (Drebrin) [3]. The Drebrins are actin binding proteins that have roles in early synaptogenesis and synaptic function through modulation of dendritic spine morphology [17,18].

While significant progress has been made in understanding the expression, function and targets of Npas4 in the adult brain, relatively little is known about its expression during embryogenesis and whether or not it may be involved in embryonic development. Only two studies have investigated the expression of Npas4 during development and the reports were inconsistent. Despite cloning the *Npas4* complementary DNA (cDNA) from a human fetal brain library, Ooe *et al*. were unable to detect *Npas4* mRNA expression in the developing mouse embryo using either *in situ* hybridization (ISH) or reverse transcription polymerase chain reaction (RT-PCR) [3]. In contrast, Hester *et al*. found Npas4 protein to be expressed in the dorsal root ganglion of the mouse embryo at embryonic day 10.5 (E10.5) [5]. The paucity of available data and the lack of a clear consensus indicate that more research is required before a definitive conclusion can be made regarding the expression of Npas4 in the embryo.

Although experimental evidence is still lacking, several lines of evidence suggest that Npas4 may have a developmental role. Firstly, it is not uncommon for bHLH PAS transcription factors to have roles in both adulthood and development. For example, in addition to regulating the hypoxic response in the adult organism [19-22], hypoxia inducible factor 1 alpha (Hif1α) is also required for proper vascularization of the embryo and, consequently, mice lacking Hif1α die *in utero* due to vascularization defects [23]. Indeed, some researchers have suggested a possible developmental role for Npas4 due to its similarity to other bHLH PAS proteins that are involved in regulating various aspects of embryonic development. Npas4 falls within a subset of bHLH PAS proteins that have a tissue-restricted expression pattern and are not dependent on pre-activation (such as ligand binding or protein stabilization) to form transcriptionally active dimers. Other factors belonging to this subgroup include the *D. melanogaster* proteins single-minded and trachealess, both of which have critical roles in development [24-26]. Moser *et al*. have proposed that since Npas4 shares a similar mode of action to these proteins it could also be important for embryonic development [2].

Secondly, consideration of evolutionarily related genes also supports the notion that Npas4 may have a role during development. The *D. melanogaster* homolog of Npas4, dysfusion (Dys), is expressed during embryogenesis and, indeed, is essential for normal development [27,28]. Given that Npas4 and Dys are descended from a common ancestral protein, it is possible that some aspects of the developmental expression or function of Dys are conserved in the mammalian Npas4 proteins.

Thirdly, several Npas4 target genes are known to be important for nervous system development. The embryonic isoform of Drebrin, Drebrin E, plays a role in axonal growth during development [29,30] while disruption of Bdnf signaling has profound consequences for neuronal survival resulting in death during the first few weeks of life [31,32].

Lastly, a computational analysis which examined networks of interacting bHLH transcription factors predicted that Npas4, together with NeuroD6, a neurogenic bHLH transcription factor important for neuronal differentiation and survival [33,34], may be part of a transcriptional regulatory module which is important in mouse brain development [35].

In this study, we used neural differentiation of embryonic stem cells (ESCs) as a model of neurogenesis to investigate the expression and function of Npas4 from a neurodevelopmental point of view. Differentiation of ESCs *in vitro* recapitulates many of the fundamental cellular and molecular events occurring during the early stages of embryogenesis [36,37] making them a useful model in which complex processes can be studied in a simplified and much more accessible way without the complexity of a whole organism. Here, we show that Npas4 is transiently up-regulated during neural differentiation of both mouse and human ESCs at a time which coincides with the proliferation of neural progenitor cells in culture. We also demonstrate that Npas4 is expressed specifically in the developing forebrain of the mouse embryo at a stage of development that is crucial for patterning of the telencephalon. Finally, we show that reduced Npas4 expression in mouse ESCs undergoing neural conversion impairs their ability to differentiate appropriately resulting in a delayed neural differentiation phenotype. Overall, our results indicate a presumptive role for Npas4 in early mammalian central nervous system (CNS) development that has been hitherto undefined.

## Materials and methods

### Ethics statement

All animals were housed and treated in accordance with the Australian Code of Practice for the Care and Use of Animals for Scientific Purposes. The University of Adelaide Animal Ethics Committee approved all experiments prior to their commencement.

### Cell culture

Cell lines used in this study were: D3 [38] and 46C [39] mouse embryonic stem cells (mESCs) and the Envy [40] human embryonic stem cell (hESC) line. Routine culture of mESCs has been described previously [41].

N2B27 differentation of mESCs: the method used for neural differentiation of mESCs as a monolayer has been described previously [42]. Briefly, mESCs were plated onto 0.1% gelatin coated dishes in N2B27 medium at a density of 1 × 10^4^ cells/cm^2^. Cells were cultured in a 37°C incubator containing a mixture of 95% air/5% CO_2_ and N2B27 medium was replaced every two days.

Noggin-induced differentiation of hESCs: the methods used for routine culture and neural differentiation of hESCs have been described previously [43]. Briefly, neural induction of hESCs was initiated by treatment with recombinant Noggin (500 ng/mL) for 14 days. To form neurospheres, Noggin treated hESC colonies were dissected and transferred to serum free medium supplemented with epidermal growth factor (20 ng/mL) and fibroblast growth factor (20 ng/mL).

### Flow cytometry

Cells were dissociated using trypsin, resuspended in PBS and the suspension was passed through a 70 μm cell strainer to remove any aggregates. Cells were then analyzed for GFP expression using a FACSCanto™ Flow Cytometer (BD Biosciences, Franklin Lakes, New Jersey, USA) and data were analyzed using FACSDiva™ (BD Biosciences, Franklin Lakes, New Jersey, USA) software. Cells were first gated to remove any cell debris or doublets and subsequently were gated to exclude the negative control D3 mESC line from the GFP positive pool - the threshold GFP fluorescence was set such that 99% of D3 cells were excluded and only cells whose fluorescence intensity was greater than this level were scored as GFP^+^.

### Immunocytochemistry

Cells were fixed in 4% paraformaldehyde (PFA) for 20 minutes at room temperature and then permeabilized using blocking solution (10% (v/v) horse serum in PBS with 0.1% Tween 20 (0.1% PBST)) for 60 minutes at room temperature. Cells were then incubated with the anti-Nestin antibody (Abcam, Cambridge, England, Ab5968; 1/800 in blocking solution) overnight at 4°C. After washing in 0.1% PBST, cells were incubated with the anti-rabbit Cy2 secondary antibody (Jackson, Pennsylvania, USA, 711-225-152; 1/200 in blocking solution) for two hours at room temperature. After washing in 0.1% PBST, cells were then incubated in 300 nM 4′,6-diamidino-2-phenylindole (DAPI) for 10 minutes at room temperature after which they were washed twice in 0.1% PBST and viewed under fluorescence microscopy. For quantification of Nestin expression, ImageJ was used to calculate the Nestin:DAPI ratio. For each field, the overall fluorescence intensity was calculated for each color channel and from this the ratio of Nestin:DAPI fluorescence was determined.

### Immunoblotting

Cells were lysed using lysis buffer (50 mM Tris–HCl pH8.0, 150 mM NaCl, 1% (v/v) NP-40, 1% (w/v) sodium deoxycholate, 0.1% (w/v) sodium dodecyl sulfate) containing protease inhibitor cocktail (Roche, Basel, Switzerland). Following sodium dodecyl sulfate polyacrylamide gel electrophoresis (SDS-PAGE), proteins were transferred onto a 0.45 μm Immobilon P PVDF membrane (Millipore™, Billerica, Massachusetts, USA) and, after blocking, the membranes were incubated overnight at 4°C with the appropriate primary antibody diluted in 0.1% PBST (Actin (Sigma-Aldrich, St. Louis, Missouri, USA, A2066; 1/5,000), GFP (Rockland, Pennsylvania, USA, 600-101-215; 1/2,500), Npas4 (kind gift from Yingxi Lin; 1/15,000)). After washing, membranes were incubated with the appropriate horse radish peroxidase (HRP)-conjugated secondary antibody diluted in 0.1% PBST (anti-goat-HRP (Rockland, 605-4302; 1/60,000), anti-mouse-HRP (Rockland, 610-703-124; 1/60,000), anti-rabbit-HRP (Rockland, 611-703-127; 1/60,000)) for two hours at room temperature. Proteins were detected using Immobilon™ Western Chemiluminescent HRP Substrate (Millipore™) according to the manufacturer’s instructions. Chemiluminescent signal intensity was quantified by densitometry analysis using ImageJ software.

### Alkaline phosphatase staining

The Alkaline Phosphatase Detection Kit (Millipore™) was used to assay for alkaline phosphatase activity according to the manufacturer’s instructions.

### Growth rate assays

Undifferentiated mESCs: cells were plated in six-well plates (9 × 10^3^ cells/cm^2^) and the number of living cells was counted at 24 hour intervals over a period of four days using the vital stain trypan blue.

N2B27 differentiation: on Day 0, ESCs were plated onto 35 mm dishes (8 × 10^4^ cells/dish) in N2B27 medium and the number of living cells was counted at 24 hour intervals over a period of ten days using the vital stain trypan blue.

### In situ hybridization

N2B27 cultures *in vitro*: The ISH probes used to detect *Npas4* mRNA expression have been used previously [1]. A 991 bp segment of Npas4 cDNA was amplified using the following primers (TATGAGAAGTTGCCCCCAAG; CGGTGAGGAAGTGAGACTCC) and was cloned into the multiple cloning site of the pGEM® T Easy vector (Promega, Fitchburg, Wisconsin, USA). The probe sequence was confirmed by sequencing using pUC/M13 primers (GTTTTCCCAGTCACGAC; CAGGAAACAGCTATGAC) and 50 ng of plasmid DNA was used as a template to amplify a linear DNA fragment containing the Npas4 probe sequence by PCR using pUC/M13 primers. PCR product (10 μg) was digested with either SacI or SacII. Digested PCR fragments were purified by agarose gel electrophoresis and excision using QIAquick® Gel Extraction Kit (QIAGEN®), Hilden, Germany according to the manufacturer’s instructions. Purified DNA (3.5 μg) was used as template to make a DIG labeled riboprobe using *in vitro* transcription. Following this, unbound DIG-11-UTP was removed using a Nanosep® Centrifugal Device (Pall Corportation, Port Washington, New York, USA) according to the manufacturer’s instructions.

Cells were fixed in 4% PFA for 20 minutes, washed twice with PBS and dehydrated through a series of ascending methanol/PBS washes (25%, 50%, 75%, 100%). Cells were immersed in 100% methanol and left overnight at 20°C and the following day cells were rehydrated with a series of descending methanol/PBS washes (75%, 50%, 25%, 0%). Cells were then permeabilized by washing in 0.1% PBST for 30 minutes after which they were again fixed in 4% PFA for 20 minutes. This was followed by three washes of five minutes each in 0.1% PBST and then one five minute wash in a 1:1 mixture of hybridization buffer (50% (v/v) formamide, 5X saline sodium citrate (SSC), 0.1% (v/v) Tween 20, 40 μg/mL salmon sperm DNA) and PBS. Subsequently, cells were incubated in hybridization buffer for 60 minutes at 65°C after which this was replaced with fresh hybridization buffer containing the DIG-labeled riboprobe (175 ng/mL) which was heated for two minutes at 80°C and then cooled for five minutes on ice prior to addition to the hybridization buffer. Hybridization was allowed to proceed overnight at 65°C in a humidified chamber. The following day, cells were washed three times in washing solution (50% (v/v) formamide, 2X SSC, 0.1% (v/v) Tween 20) for 30 minutes at 65°C and then twice in maleic acid buffer (0.1 M maleic acid, 0.15 M NaCl) for 30 minutes. After this, cells were incubated in blocking solution (1% (w/v) blocking reagent (Roche, 11-096-176-001), 10% (v/v) horse serum in maleic acid buffer) for 60 minutes after which this was replaced with fresh blocking solution containing the anti-DIG antibody (Roche, 11-093-274-910; 1/5,000). Cells were incubated with the anti-DIG antibody overnight at 4°C and the following day were washed three times in maleic acid buffer for 20 minutes, followed by two washes of 10 minutes each in detection buffer (0.1 M Tris-Cl, 0.1 M NaCl). Coverslips were placed face up onto a microscope slide and detection solution (2% (v/v) NBT/BCIP stock solution (Roche, 11-681-451-001) in detection buffer) was added to the slides (500 μL/slide) and the color reaction was allowed to develop overnight at room temperature in the dark. Once color development was complete, cells were washed three times in PBS, fixed in 4% PFA for 20 minutes and washed twice more in PBS. Following this, coverslips were placed upside down onto a drop of mounting medium which has been placed onto a microscope slide. The mounting medium was allowed to cure overnight and the following day slides were viewed microscopically.

Whole-mount mouse embryo: After embryos were isolated, they were washed three times in PBS and dehydrated through a series of ascending methanol/0.1% PBST washes (25%, 50%, 75%, 100%) of five minutes each. Embryos were immersed in 100% methanol (2x five minutes), left overnight at 20°C and the following day were rehydrated with a series of descending methanol/PBS washes (75%, 50%, 25%, 0%) of five minutes each. Endogenous hydrogen peroxide activity was then quenched by incubating embryos in 6% H_2_O_2_ in PBT for one hour at room temperature. Embryos were then permeabilized with Proteinase K (Cat. # P6556. Sigma-Alrich (St. Louis, Missouri, USA) (10 μg/mL in PBT) treatment (25 minutes at room temperature for E8.0, 30 minutes for E9.5 and 35 minutes for E10.5). Proteinase K activity was arrested by washing for five minutes at room temperature with fresh glycine (2 mg/mL in PBT). Embryos were then re-fixed in 0.2% gluteraldehyde/4% PFA in PBT for 20 minutes at room temperature. Embryos were then washed twice for five minutes in PBT and rinsed once in a 1:1 mixture of PBT:hybridization solution (hybridization solution: 50% formamide, 5X SSC, 1% SDS, 50 μg/mL heparin, 50 μg/mL yeast RNA, 0.1% Tween20 in RNase-free water). Embryos were then rinsed in hybridization solution before being incubated in hydridization solution for four hours at 70°C with rotation. After blocking, the solution was replaced with hybridization solution containing DIG-labeled Linked Nucleic Acid (LNA™, Exiqon (Copenhagen, Denmark)) probes diluted to 20 nM (Npas4 TGATCTGCTGAATGAGACGAT; Scrambled GTGTAACACGTCTATACGCCCA, Exiqon 300512–15) and hybridization was allowed to proceed overnight at 57°C with rotation. The following day, embryos were washed twice in pre-heated Solution #1 (50% formamide, 4X SSC, 1% SDS in water) for 30 minutes at 70°C. Next, embryos were washed once in a 1:1 mixture of Solution #1:Solution #2 (0.5 M NaCl, 10 mM Tris pH7.5, 0.1% Tween20 in water) for 10 minutes at 70°C. Embryos were washed three more times in Solution #2 for five minutes at room temperature. Unbound probe was then degraded by incubating embryos twice in RNase A (100 ug/mL in Solution #2) for 30 minutes at 37°C. Embryos were then washed once in Solution #2 for five minutes at room temperature, once in Solution #3 (50% formamide, 2X SSC, in water) for five minutes at room temperature, twice in Solution #3 for 30 minutes at 65°C and three times in Tris buffered saline with 0.1% Tween20 (TBST) for five minutes at room temperature. Embryos were then blocked by incubating for 1.5 hours in 10% heat-inactivated sheep serum in TBST. Following this, the blocking solution was replaced with fresh blocking solution containing the anti-DIG antibody (Roche, 11-093-274-910; 1/2,000) and embryos were incubated overnight at 4°C with rocking. The following day, embryos were washed three times for five minutes in TBST at room temperature, then five times for one hour in TBST at room temperature and then overnight in TBST at 4°C with rocking. The following day, embryos were washed three times in NTT solution (0.1 M NaCl, 0.1 M Tris pH9.5, 0.1% Tween20) for 10 minutes at room temperature. To develop the color reaction, embryos were incubated at 4°C and protected from light in NTT solution containing NBT/BCIP stock solution (Roche, 11681451001, 1/50) for several days until staining was visible. Embryos were then washed twice for 10 minutes in NTT solution before being washed overnight in PBT at room temperature. The following day, embryos were fixed in 0.2% glutaraldehyde/4% PFA for one hour at room temperature, washed twice for five minutes in PBS and then transferred to 80% glycerol for imaging.

### Generation of stable Npas4 knockdown mESC lines by lentiviral transduction

MISSION® shRNA constructs (Sigma-Aldrich: pLKO.1-puro control vector, SHC001; Npas4, SHGLY-NM_153553) were used to generate Npas4 knockdown and control lentivirus. 46C mESCs were transduced and clones were isolated using antibiotic selection (3 μg/mL puromycin for 10 days. Although the 46C mESC line does already inherently carry the puromycin N-acetyl-transferase gene within its genome, it is under the control of the endogenous Sry-related high mobility group box 1 (Sox1) promoter and, therefore, is not actively expressed in undifferentiated mESCs. Thus, we were able to isolate transduced cells harboring integrated constructs using puromycin selection while they remained undifferentiated. The sequences targeted by the Npas4 knockdown constructs are as follows; Npas4 KD1 - CCTGGATCTTAAACCCTGGAA, Npas4 KD2 - CGTTTCTGAAAGTGTCCTAAT.

### Nucleic acid isolation

Isolation of genomic DNA (gDNA) was performed using the DNeasy Blood and Tissue Kit (QIAGEN) according to the manufacturer’s instructions. RNA was isolated using the High Pure RNA Isolation Kit (Roche) according to the manufacturer’s instructions. Included in the protocol is a DNaseI treatment step to remove traces of gDNA.

### Reverse transcription

RT was performed using the SuperScript™ III reverse transcriptase enzyme (Invitrogen™, Carlsbad, California, USA) according to the manufacturer’s instructions.

### Polymerase chain reaction

Primer sequences and PCR conditions are described in detail in Additional file 1.

### Quantitative RT PCR

Quantitative RT PCR (qRT-PCR) reactions were performed using SYBR® Green PCR Master Mix (Applied Biosystems®, California, USA) according to the manufacturer’s instructions using 50 ng of template cDNA. Samples were loaded in triplicate and thermal cycling was performed using an ABI PRISM® 7000 Sequence Detection System driven by ABI PRISM SDS v1.1 software (Applied Biosystems®). β-actin was used as an internal reference to facilitate relative quantification using the comparative Ct method [44]. Primers directed to the Npas4 transcript were similar in reaction efficiency to the β-actin internal reference primers.

### Mouse embryo collection

Uteri of pregnant mice were collected at the indicated times and the embryos dissected from the decidua in cold PBS. For ISH experiments, the C57BL6 strain was used and embryos were immediately fixed in 4% PFA overnight at 4°C, while for RT-PCR experiments the B6CBAF1 strain was used and embryos from the same litter were pooled and RNA extracted immediately.

## Results

### *Npas4* is transiently expressed during neural differentiation of ESCs

**Figure 1:**
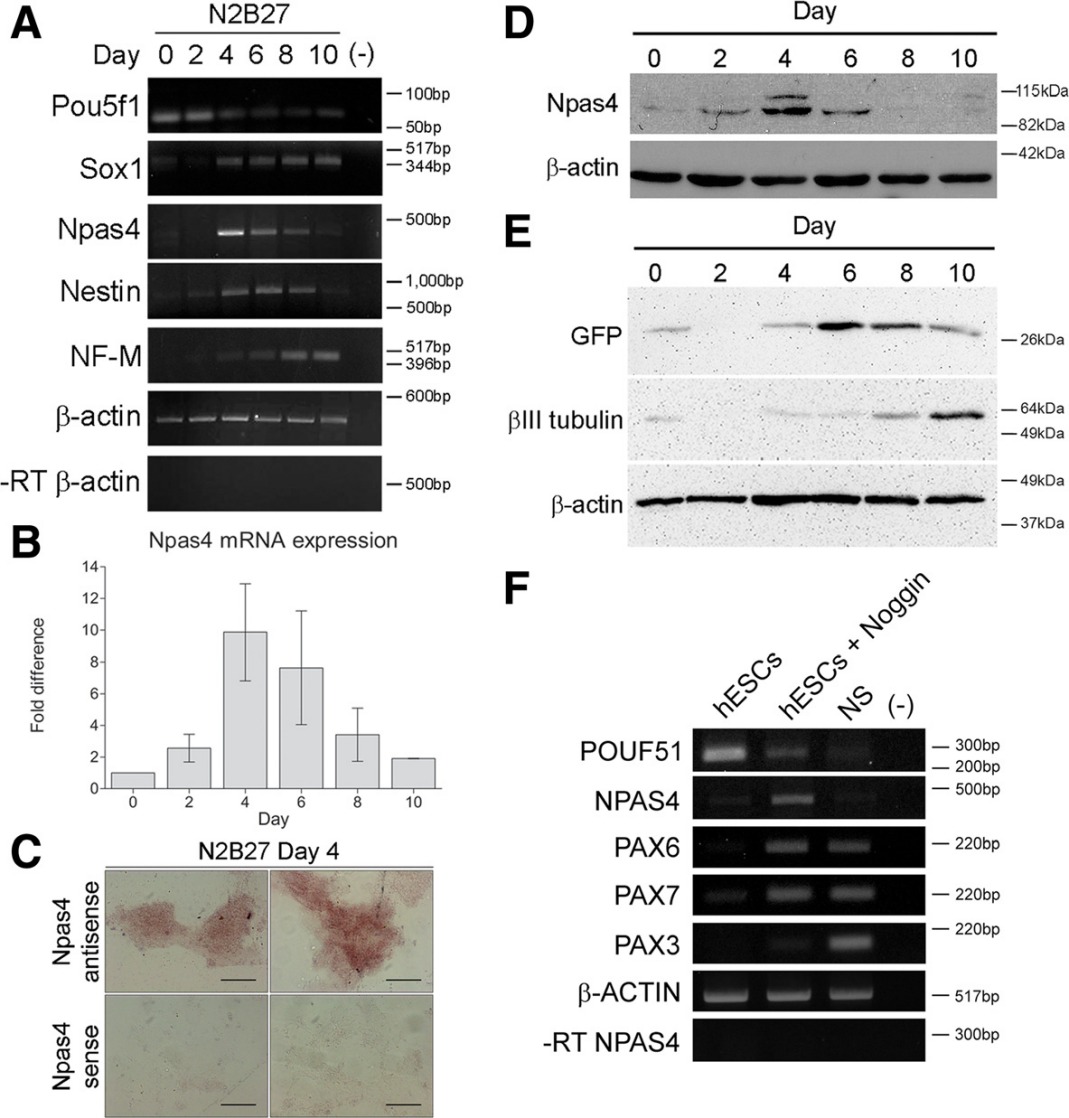
***Npas4 *expression during neural differentiation of ESCs. (A)** RT-PCR analysis was used to determine the temporal expression profile of *Npas4* mRNA in relation to various marker genes during N2B27 differentiation of mESCs (n = 3). Primers to the reference gene *β-actin* were used as a loading control. The negative control reaction (−) contained water in place of template cDNA. **(B)** The changes in *Npas4* expression during N2B27 differentiation were quantified using qRT-PCR. At each time point *Npas4* expression was normalized to *β-actin* expression and fold changes are relative to Day 0 (undifferentiated mESCs). Mean values and standard deviations of three independent experiments (n = 3) are displayed. **(C)***In situ* hybridization analysis of *Npas4* mRNA expression at Day 4 of N2B27 differentiation of mESCs. Representative images of differentiating colonies from two independent experiments (n = 2) are shown. Top panel - *Npas4* antisense probe; Bottom panel - *Npas4* sense probe. Scale bar = 100 μm. **(D)** Immunoblotting was used to determine the temporal expression profile of the Npas4 protein during N2B27 differentiation of mESCs. An antibody to the reference protein β-actin was used as a loading control (n = 3). **(E)** Immunoblotting was used to determine the temporal expression profile of Sox1 in the 46C cell line (using an antibody to GFP) and βIII tubulin during N2B27 differentiation of mESCs. An antibody to the reference protein β-actin was used as a loading control (n = 3). **(F)** RT-PCR analysis of *NPAS4* mRNA in relation to various marker genes during Noggin-induced neural differentiation of hESCs (n = 3). Primers to the reference gene *β-ACTIN* were used as a loading control. The negative control reaction (−) contained water in place of template cDNA. hESCs, human embryonic stem cells; mESCs, mouse embryonic stem cells; NS, neurospheres.

To characterize the temporal expression profile of *Npas4* during neural differentiation of mouse ESCs (mESCs), 46C mESCs were differentiated using the N2B27 method [42] and RT-PCR was used to assay for *Npas4* mRNA expression at 48 hour intervals over the course of ten days. Undifferentiated mESCs (Day 0) were also analyzed to determine whether *Npas4* is expressed by pluripotent mESCs. To place the *Npas4* expression profile in context, the expression of various marker genes was also examined including genes that are expressed by pluripotent ESCs (*Pou5f1*), neural progenitor cells (NPCs) (*Sox1*, *Nestin*) and neurons (*NF-M*). We observed that *Npas4* was transiently up-regulated during neural differentiation with expression commencing at approximately Day 4, once expression of Pou5f1 was down-regulated, and declining at around Day 8 just prior to the onset of NF-M expression (Figure 1A). In this way, the expression pattern of *Npas4* was similar to that of the NPC marker gene Nestin. When the change in *Npas4* expression was quantified using qRT-PCR, we found that the highest expression of *Npas4* occurred at Day 4 when a 10-fold induction was observed relative to undifferentiated mESCs (Figure 1B). We then investigated the spatial expression pattern of *Npas4* during neural differentiation of mESCs using ISH. After four days of differentiation, a strong signal was observed in virtually all colonies with most of the cells in each colony showing staining (Figure 1C). In negative control experiments where a sense probe was used, no signal was detected in any colonies.

Next, Npas4 protein expression during N2B27 differentiation of mESCs was investigated using immunoblotting. Similar to the mRNA profile, Npas4 protein expression was transient reaching a peak at around Day 4 of differentiation (Figure 1D). The specificity of the anti-Npas4 antibody was validated by transfecting HEK 293 T cells, which do not express Npas4 protein endogenously, with an Npas4 expression construct (Additional file 2). Once again, expression of various marker genes was also investigated to place Npas4 protein expression in context. The 46C mESC line [39] is a transgenic GFP knock-in cell line in which the open reading frame of the *Sox1* gene has been replaced with a dual reporter/selection cassette containing the sequence coding for enhanced GFP followed by an internal ribosome entry site (IRES)-linked puromycin resistance gene such that both genes are under the control of the endogenous Sox1 promoter and are expressed concurrently in Sox1^+^ cells. Thus, using the 46C mESC line we were able to indirectly assess expression of Sox1 by using an antibody to GFP or by using fluorescence microscopy. GFP expression was present at low levels after four days of differentiation, peaked between Days 6 and 8 and then gradually declined from Day 8 onwards (Figure 1E, Additional file 3). In contrast, the neuronal marker βIII tubulin was not expressed at significant levels until around Day 8 (Figure 1E). Thus, Npas4 protein expression overlapped slightly with that of Sox1 but was down-regulated prior to the onset of βIII tubulin expression.

To determine whether the pattern of *Npas4* expression observed during neural differentiation of mESCs was conserved in other mammalian species, the temporal profile of *NPAS4* expression was also investigated during Noggin-induced neural differentiation of hESCs using RT-PCR. Samples corresponding to three stages of differentiation were analyzed; undifferentiated hESCs, hESCs treated with Noggin for 14 days and neurospheres derived from Noggin-treated hESCs that had been cultured for 19 days. Once again, expression of well-characterized marker genes was used as a reference point; these included markers of pluripotent ESCs (*POU5F1*), early NPCs (*PAX6*, *PAX7*) and late NPCs (*PAX3*).

As in the mESC model, we found that *NPAS4* mRNA expression was transiently up-regulated during neural differentiation of hESCs with *NPAS4* mRNA expression being detected specifically in Noggin-treated hESCs, but absent in undifferentiated hESCs or neurospheres (Figure 1F). This was different from the expression of NPC marker genes *PAX6* and *PAX7,* which were expressed in both Noggin-treated hESCs and neurospheres.

### Npas4 is expressed in the developing mouse embryo

**Figure 2:**
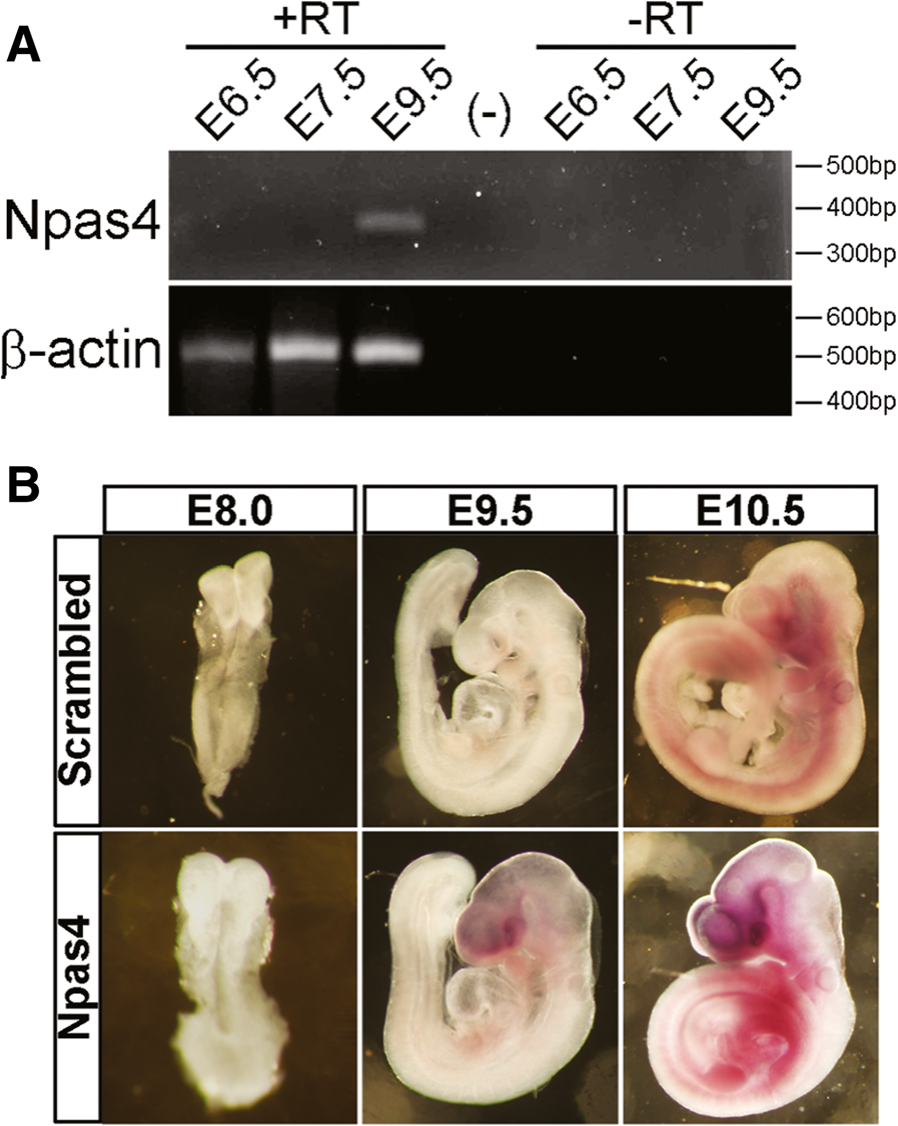
***Npas4 *mRNA expression during mouse embryonic development. (A)** RT-PCR analysis was used to determine *Npas4* mRNA expression at various stages of mouse embryonic development ranging from E6.5 to E9.5. RNA was isolated from mouse embryos at the indicated developmental stage and this was used as a template for reverse transcription in a reaction containing either the reverse transcription enzyme (+RT) or water (-RT). The PCR negative control reaction (−) contained water in place of template cDNA. Primers to the reference gene *β-actin* were used as a positive control for cDNA synthesis. **(B)***In situ* hybridization was performed on whole-mount mouse embryos at E8.0, E9.5 and E10.5 using a DIG-labeled LNA probe that was either complementary to the *Npas4* mRNA (Antisense) or was a non-specific control probe (Scrambled). E, embryonic day.

Given that *Npas4* was found to be transiently up-regulated in two independent *in vitro* models of neural differentiation, the expression of *Npas4* was investigated in the whole mouse embryo to determine whether a similar pattern of expression also occurs at an equivalent stage during murine embryonic development *in vivo*. RNA was extracted from whole embryos and RT-PCR was used to analyze the temporal profile of *Npas4* mRNA expression at various stages of embryogenesis ranging from E6.5 to E9.5 (Figure 2A). Using this method, we were able to detect Npas4 expression at E9.5 but not at the earlier time points of E7.5 or E6.5. To validate these findings and to determine the spatial expression pattern of Npas4 in the embryo, we performed whole-mount ISH using an Npas4-specific DIG-labeled LNA probe on embryos of various stages of development from E8.0 to E10.5 (Figure 2B). As a negative control, we used a scrambled LNA probe which does not recognize the *Npas4* mRNA sequence. Confirming our previous observations, we did not detect any Npas4 expression prior to E9.5; however, at E9.5 Npas4 expression was observed exclusively in the prosencephalon. At E10.5 this expression had intensified and expanded caudally. Thus, Npas4 is expressed in the developing nervous system of the mouse embryo at a time which coincides with the onset of neurulation and patterning of the telencephalon.

### Knockdown of Npas4 expression during neural differentiation of 46C mESCs

**Figure 3:**
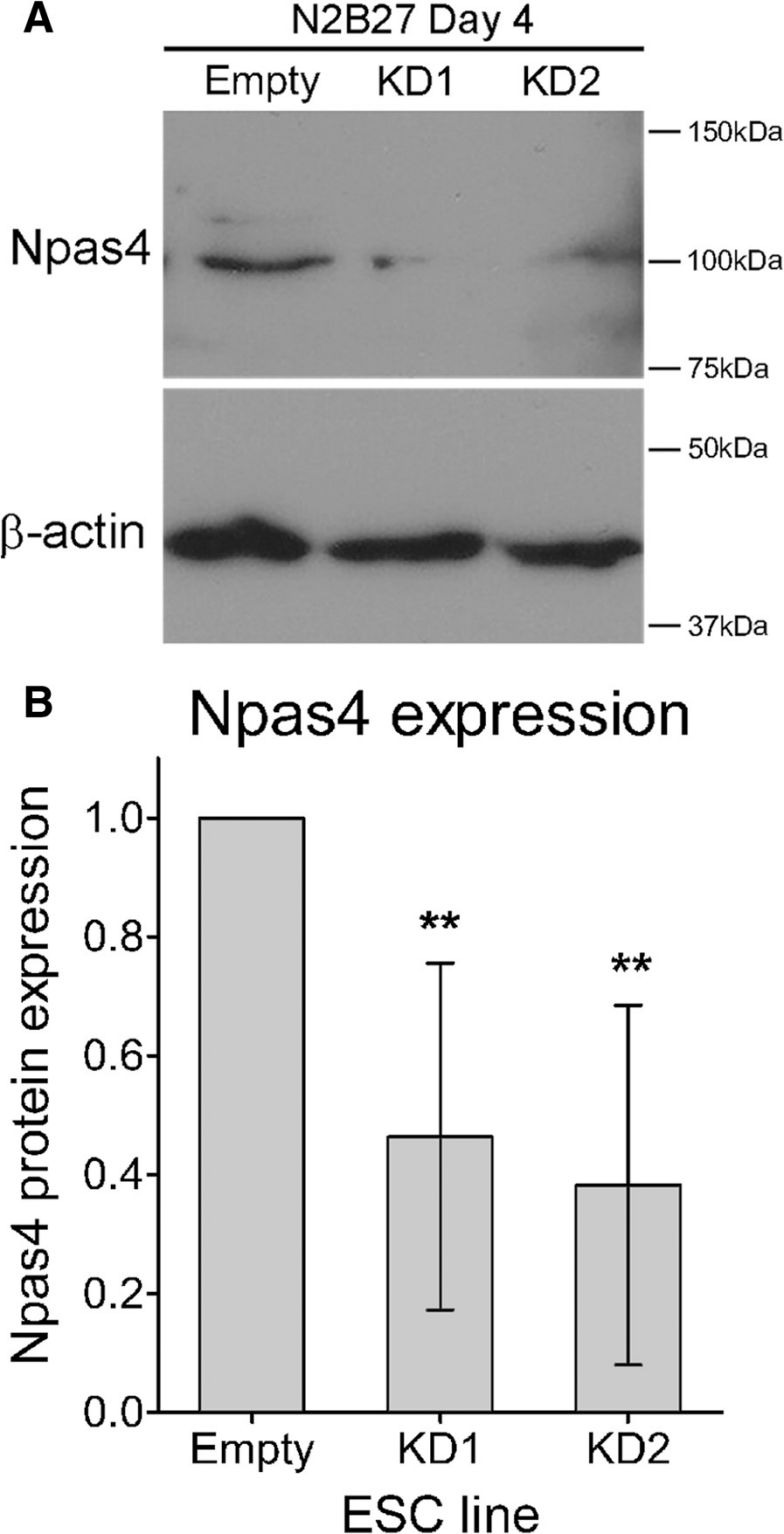
**Knockdown of endogenous Npas4 protein during neural differentiation of mESCs. (A)** Immunoblotting was used to determine the level of Npas4 protein expression in different mESC lines (n = 3 biological replicates). Lysates were harvested after four days of differentiation in N2B27 medium and blots were probed with an anti-Npas4 antibody. β-actin served as a loading control. **(B)** Npas4 knockdown was quantified by densitometry analysis. Npas4 protein expression in each sample was normalized to β-actin expression and is relative to Npas4 expression in the Empty vector control line which was given an arbitrary value of 1. Mean values and standard deviations of three independent experiments (n = 3 biological replicates) are shown (**P <0.01, one-way ANOVA). ANOVA, analysis of variance; mESC, mouse embryonic stem cells.

To investigate the role of Npas4 during neural differentiation, RNA interference (RNAi) was used to reduce Npas4 expression in mESCs undergoing differentiation. Given the pattern of Npas4 expression during embryonic development and neural differentiation of ESCs, the 46C mESC line was selected as the parental cell line for Npas4 knockdown experiments to facilitate detection of possible phenotypes affecting Sox1 expression and/or NPC formation. Lentiviral transduction was used to introduce small hairpin RNA (shRNA) expression constructs into 46C mESCs and antibiotic selection was used to generate stable, clonally-derived Npas4 knockdown mESC lines. Two different Npas4 shRNA constructs were used to create Npas4 knockdown mESC lines (KD1 and KD2) and, in addition, an Empty vector control mESC line was created to control for the transduction process and for the introduction of foreign DNA into cells. Immunoblotting was used to assess Npas4 expression in the transduced mESC lines after four days of neural differentiation. Expression of the endogenous Npas4 protein was reduced in both the Npas4 knockdown mESC lines when compared to the Empty vector control (approximately 54% reduction in KD1 and 62% reduction in KD2) (Figure 3A) and, when analyzed using a one-way analysis of variance (ANOVA), this difference was statistically significant (*P* <0.001) (Figure 3B). In the undifferentiated state, all of the transduced mESC lines retained normal growth rates, morphology and expression of several key pluripotency genes (Additional file 4) suggesting that the genomic integration of shRNA constructs had not interfered with normal ESC biology and that interpretation of results would not be affected by off-target integration effects. Furthermore, no difference was observed between the Empty vector control mESC line and those constitutively expressing Npas4-specific shRNAs suggesting that Npas4 is dispensable for normal ESC biology.

### Reduced Npas4 expression during neural differentiation results in delayed neural differentiation

**Figure 4:**
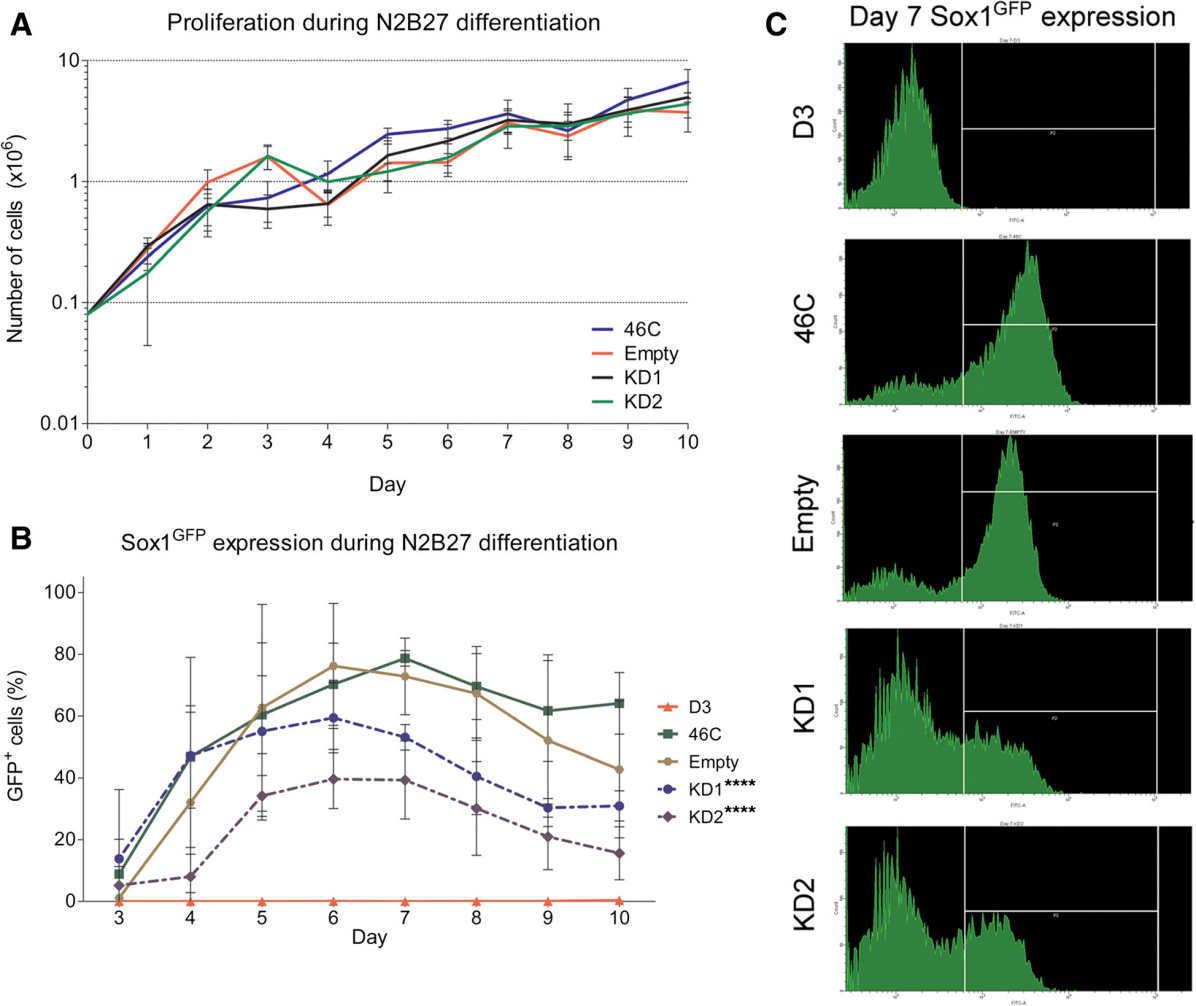
**The effect of reduced Npas4 expression on Sox1 expression during neural differentiation of mESCs. (A)** The cell proliferation rate during neural differentiation was measured by counting the total number of viable cells at 24 hour intervals. Means and standard deviations of three independent experiments are shown (n = 3). There was no statistically significant difference between the Npas4 knockdown lines and the Empty vector control mESC line (*P* = 0.2739, non-linear regression). **(B)** Temporal analysis of Sox1^GFP^ expression during N2B27 differentiation of Npas4 knockdown and control mESCs as measured by flow cytometry. Means and standard deviations of three independent experiments are shown (n = 3). There was a statistically significant difference between each of the Npas4 knockdown mESC lines and the Empty vector control mESC line (****P ≤0.0001, two-way ANOVA). There was no statistically significant difference between the Empty vector control mESC line and the parental 46C mESC line (*P* = 0.1898 , two-way ANOVA). **(C)** Sox1^GFP^ expression at Day 7 of neural differentiation. Representative flow cytometry histograms are shown for each mESC line. The y-axis shows the number of cells counted while the x-axis shows the GFP fluorescence intensity. The gate used to count GFP^+^ cells is shown. ANOVA, analysis of variance; mESC, mouse embryonic stem cells.

To determine whether reduced Npas4 expression affected neural differentiation of mESCs, the Npas4 knockdown mESC lines were compared to the Empty vector control mESC line in their ability to undergo neural differentiation. We first assessed the proliferation rate of the Npas4 knockdown lines to determine whether knockdown of Npas4 resulted in a gross change in proliferation or cell death during neural differentiation. We compared the proliferation rate of Npas4 knockdown mESC lines to that of the Empty vector control mESC line by counting the total number of viable cells at 24 hour intervals over ten days of differentiation for each cell line. We found that there was no statistically significant difference in proliferation rate between the Npas4 knockdown lines and the Empty vector control mESC line when analyzed using non-linear regression, *P* = 0.2739 (Figure 4A).

The distinctive temporal expression profile of Npas4 during neural differentiation of mESCs (Figure 1A, 1B, 1D) suggested that Npas4 may be expressed by a transient progenitor cell type as the period of peak Npas4 expression corresponded to the phase of differentiation marked by the proliferation of NPCs [39]. Thus, to determine the effect of Npas4 knockdown on NPCs formation, flow cytometry was used to evaluate the occurrence of Sox1^+^ NPCs in both Npas4 knockdown and control mESCs during neural differentiation. To ensure that background auto-fluorescence did not interfere with detection of Sox1^GFP^ expression, the D3 mESC line [38], which does not carry the gene coding for GFP in its genome, was used as a negative control.

The Sox1^GFP^ expression profile of the Empty vector control mESC line was comparable to that of the 46C parental mESC line and no statistically significant difference was detected when the two cell lines were compared using two-way ANOVA, *P* = 0.1898 (Figure 4B). In both cell lines, the proportion of GFP^+^ cells steadily increased between Days 3 and 5 reaching a peak of approximately 80% GFP^+^ cells between Days 6 and 7 before gradually declining. Analysis of Npas4 knockdown mESC cultures revealed that the Sox1^GFP^ expression profiles for these cell lines had a similar pattern to those of the control mESC lines although the maximum percentage of GFP^+^ cells attained was considerably lower. The highest percentage of GFP^+^ cells recorded in KD1 cultures was 60%, while in KD2 cultures this figure was only 40%. Over the course of the differentiation period, the difference in the percentage of GFP^+^ cells generated between the Empty vector control mESC line and each of the Npas4 knockdown mESC lines was statistically significant when tested using a two-way ANOVA (*P* <0.0001). Representative histogram plots of GFP fluorescence intensity recorded on Day 7 of differentiation are shown in Figure 4C. These data suggest that reduced Npas4 expression during neural differentiation of mESCs resulted in a decrease in the proportion of cells expressing Sox1.

**Figure 5:**
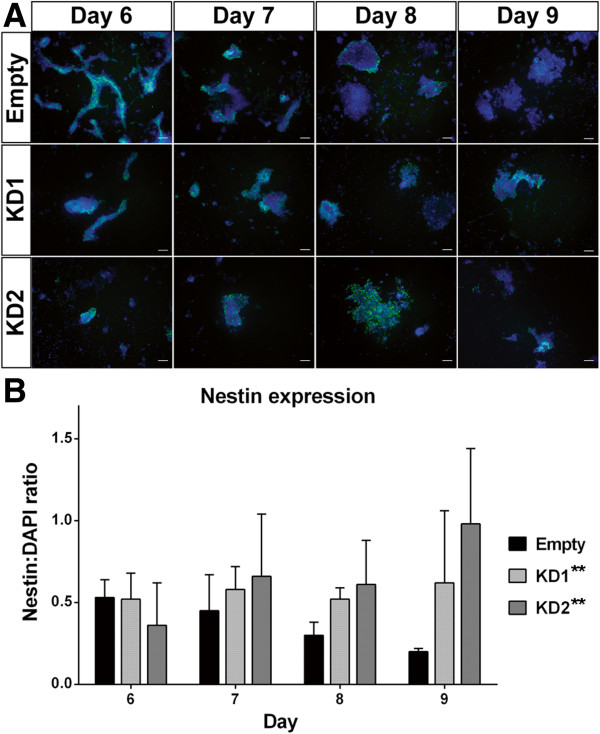
**The effect of reduced Npas4 expression on Nestin expression during neural differentiation of mESCs. (A)** Immunocytochemical analysis of Nestin expression (green) during neural differentiation. Representative images of cultures are shown for each mESC line at Day 6 to 9 of N2B27 differentiation. Cells were counterstained with DAPI to visualize nuclei (blue). Scale bar = 100 μm. **(B)** Quantification of Nestin expression in Npas4 knockdown and control mESC lines at various stages of neural differentiation. For each field, the overall fluorescence intensity was calculated for each color channel and from this the ratio of Nestin:DAPI fluorescence was determined. Means and standard deviations of three independent experiments are shown (n = 3). Over the course of the differentiation period, there was a statistically significant difference in the Nestin:DAPI ratio between the Empty vector control line and each of the Npas4 knockdown lines (Empty versus KD1, **P <0.01; Empty versus KD2, **P <0.01) when analyzed using a two-way ANOVA. ANOVA, analysis of variance; DAPI, 4*'*,6-diamidino-2-phenylindole; mESC, mouse embryonic stem cells.

We next analyzed Npas4 knockdown and Empty vector control cultures for expression of another NPC marker, Nestin, using immunohistochemistry. The *Nestin* gene encodes an intermediate filament protein whose expression is specific to CNS progenitor cells [45]; however, in the embryonic CNS Nestin is expressed at a later stage of differentiation than Sox1 [46] and thus Nestin^+^ cells represent a more mature type of NPC. After four days of differentiation, no statistically significant difference was observed between mESC lines in the percentage of cells expressing Nestin (Additional file 5). However, during the later stages of differentiation a clear trend emerged. From Day 6 to Day 9, there was a steady decline in the proportion of Nestin^+^ cells present in Empty vector control cultures as these NPCs differentiated into post-mitotic neurons, which was in contrast to the Npas4 knockdown cultures where the proportion of Nestin^+^ cells remained constant (KD1) or even increased (KD2) over the same period (Figure 5). Over the course of this four day differentiation period, there was a statistically significant difference in the Nestin:DAPI ratio between the Empty vector control line and each of the Npas4 knockdown lines (Empty versus KD1, **P <0.01; Empty versus KD2, **P <0.01) when analyzed using a two-way ANOVA. These data indicate that Nestin remains expressed for a longer period of time when Npas4 expression is reduced which suggests that neural differentiation may be stalled or delayed in Npas4 knockdown cultures.

## Discussion

The possible role of Npas4 during neurodevelopment and neuronal differentiation is a topic that has received little attention to this point, despite there being evidence that it may indeed be involved in these processes. Yun *et al.* showed that *Npas4* mRNA is up-regulated during neuronal differentiation of the Neuro2a neuroblastoma cell line and that Npas4 plays an important role in promoting neurite outgrowth in both Neuro2a cells and hippocampal neurons [47]. Here, we expand on these observations and demonstrate for the first time that Npas4 is also expressed during neural differentiation of both mouse and human ESCs and that it may play a role in the progression of neural progenitor cells along the differentiation route. In both neural differentiation models, *Npas4* expression was transient and occurred prior to the emergence of neurons in culture. We speculate that Npas4 expression occurs in an early NPC population that exists transiently prior to terminal differentiation into post-mitotic neurons and glia. This assertion is supported by data from the hESC model where *NPAS4* was selectively expressed in Noggin-treated hESCs but was down-regulated once cells had progressed to the neurosphere stage. Treatment of hESCs with Noggin converts them into a population of committed early NPCs; after 14 days of treatment the majority of cells in culture are PAX6^+^/SOX1^−^ cells with very few PAX6^+^/SOX1^+^ cells present [43]. These PAX6^+^/SOX1^−^ NPCs cells have a distinct anterior identity and have been referred to as ‘primitive anterior neurectoderm’ (PAN) [48]. Replating the Noggin-treated cells triggers further differentiation and formation of neurospheres, which represent a more mature type of NPC population that also expresses PAX3. We hypothesize that during neural differentiation Npas4 is expressed by the PAN cell population and this is supported by the specific expression of Npas4 that is seen in the developing forebrain of the mouse embryo. Interestingly, it has been reported that a small number of Npas4^+^ cells in the dentate gyrus of adult mice co-express markers of dividing cells such as Sox2 and doublecortin [49] which suggests that expression of Npas4 in NPCs is not limited to the developmental period.

We also demonstrate that reduced Npas4 expression in mESCs undergoing neural induction resulted in delayed neural differentiation. In these cultures we found that there were fewer Sox1^+^ cells and that expression of Nestin persisted longer than in control cultures. Interestingly, in both cases we observed a more pronounced effect in the cell line having a greater reduction in Npas4 expression. This suggests that the severity of the phenotype is proportional to the level of Npas4 knockdown. Given that we have achieved only partial knockdown of Npas4 expression in this system, it is possible that an even more significant effect would be observed by further reducing Npas4 levels or by using mESCs obtained from Npas4^−/−^mice.

Differentiation of ESCs is commonly used to model the events occurring in the embryo during the early stages of development [36,37]. Accordingly, our observations *in vitro* suggested that Npas4 may be involved in aspects of early nervous system development *in vivo*. This was substantiated by our analysis of *Npas4* expression in the mouse embryo where we detected mRNA expression in the developing forebrain at E9.5 and E10.5. No expression was observed at the earlier stage of E8.0 and as later embryonic stages were not investigated it is not known whether Npas4 expression is maintained until birth or is only transiently expressed as was seen *in vitro*. More research is needed to address this question.

Curiously, the temporal relationship between Npas4 and Sox1 expression differed in the ESC differentiation models as compared to the mouse embryo. While Npas4 appears to be expressed prior to or concurrently with Sox1 in the *in vitro* differentiation systems, this seemed to be reversed *in vivo*. During embryogenesis Sox1 expression coincides with the neural induction of ectoderm at E7.5 [50] when it is expressed specifically in the neural plate [51] and it continues to be expressed in the neural tube along the entire anterior/posterior axis at later stages of neurodevelopment [50]. This places Sox1 expression several days ahead of Npas4 expression which we were unable to detect prior to E9.5 (Figure 2). While this seems to contradict the *in vitro* data, it should be noted that differentiation of ESCs *in vitro* is not a perfect model of embryonic development and that some differences may exist between the simplified *in vitro* model and the complexity of the whole embryo.

Nevertheless, these observations suggest that our loss-of-function studies *in vitro* may have relevance *in vivo*. The period between E7.5 to E10.5 is a critical time in the embryo when development of the nervous system begins and it is marked by several important events. At E7.5, secretion of diffusible dorsalizing signals, such as Noggin, triggers neural induction in which a region of the ectoderm becomes specifically defined as the neuroectoderm [52]. By E8.5, neural specification is largely complete and the resulting neural plate begins to fold onto itself to form the neural tube in a process known as neurulation which terminates at around E10 with the final closure event [53]. The timing of Npas4 expression and its specific localization in the rudimentary forebrain structures raise the possibility that it may be involved in the specification of forebrain identity. However, unravelling the role of Npas4 in embryogenesis and determining precisely which cell populations express this transcription factor will require further investigation. A thorough exploration of the developmental biology of Npas4-null embryos, particularly the expression patterns of Sox1 and Nestin, may provide valuable information towards this end.

While the findings from this study suggest that Npas4 could have a role in early developmental processes, the study of Npas4^−/−^mice has thus far not revealed any clear embryological function for Npas4. Npas4^−/−^mice do not show any gross morphological abnormality at birth and they are able to generate offspring in the expected Mendelian ratios [7,9]. While these observations demonstrate that the *Npas4* gene is not essential for embryonic survival, we would suggest that, based on our results, further detailed investigation of Npas4^−/−^mice during the early stages of embryogenesis is warranted. There are numerous examples of genes that have roles in important developmental processes but do not show a profound developmental phenotype when deleted, particularly when there may be compensation by other genes having similar functions. For instance, the *Sox1* gene is specifically expressed in the neural plate during early CNS development [51] and is involved in maintenance of NPCs [46,54], yet Sox1^−/−^mice are viable, born in the expected Mendelian ratios and show no obvious signs of developmental abnormalities [55].

Indeed, there is evidence to suggest that alternative pathways are activated in Npas4^−/−^mice to compensate for the lack of Npas4 protein. For example, Lin and colleagues remarked that although frequencies of miniature inhibitory post-synaptic currents (mIPSCs) were similar in hippocampal slices prepared from Npas4^+/+^and Npas4^−/−^mice, a clear difference was seen between Npas4 knockdown and controls when RNAi was used to acutely reduce Npas4 expression in wildtype slices [7]. Similarly, Ramamoorthi *et al.* noted that while there was reduction in the expression of certain Npas4 target genes in Npas4 global knockout mice, conditional deletion of Npas4 in adult mice resulted in an even more pronounced reduction in target gene expression [8]. Both of these observations imply that the presence or absence of Npas4 during embryonic development can influence which developmental programs are activated. A complete absence of Npas4 during embryonic development appears to trigger various Npas4-independent compensatory pathways that allow embryonic development to proceed relatively normally such that the resulting animal can function without the need for Npas4 activity. On the other hand, it seems that in wildtype animals, where Npas4 is an integral part of development, the neural pathways that are produced are reliant on Npas4 function and the sudden removal of Npas4 from the system has devastating effects. Presumably, this type of gene compensation is more likely to occur in the intact organism where there are many feedback mechanisms in place to ensure that development can proceed normally. In contrast to the complex environment that is an embryo, a simplified two-dimensional cellular model of development that lacks these sophisticated feedback mechanisms is likely to be much more susceptible to perturbations in gene expression. This may explain why sometimes certain phenotypes can be elicited by gene knockdown *in vitro*, yet they are not detectable in the knockout embryo.

In mESCs cultures having reduced levels of Npas4 we observed that expression of Sox1 was affected which raises the possibility that Npas4 acts upstream of Sox1 and may participate in the regulation of Sox1 expression. If Npas4 does indeed influence expression of Sox1, then it would be expected that in the Npas4^−/−^mouse there would be some evidence of disrupted Sox1 function. Interestingly, there is some similarity between the Npas4^−/−^and Sox1^−/−^mice; both knockout mice display a hyperexcitability phenotype. One of the features of the Sox1^−/−^phenotype is enhanced synaptic activity in the olfactory cortex which manifests as spontaneous epileptiform discharges and ultimately results in spontaneous limbic seizures beginning at four to six weeks of age [55,56]. Given that Npas4^−/−^mice display a similar hyperexcitability phenotype and are also prone to seizures [7], it is tempting to speculate that there may be a common underlying mechanism. In summary, our work highlights a potential role for Npas4 in neural differentiation during CNS development.

## Conclusions

Our findings provide the first evidence that Npas4 is expressed by a progenitor cell type in the context of mammalian development and that Npas4 expression is required for the normal progression of neural progenitor cells along the differentiation route. While these findings suggest that Npas4 may be involved in aspects of NPC biology, such as NPC maintenance and/or differentiation, further research is needed to confirm the developmental role of Npas4 *in vivo*.

## Abbreviations

ANOVA: analysis of variance; Bdnf: brain-derived neurotrophic factor; bHLH PAS: basic helix-loop-helix Per-Arnt-Sim; bp: base pair; CNS: central nervous system; DAPI: 4*'*,6-diamidino-2-phenylindole; E: embryonic day; ESC: embryonic stem cell; GFP: green fluorescent protein; hESC: human ESC; HRP: horseradish peroxidase; ISH: *in situ* hybridization; mESC: mouse ESC; Npas4: neuronal PAS domain protein 4; NPCs: neural progenitor cells; PAN: primitive anterior neuroectoderm; PBS: phosphate-buffered saline; PBST: PBS with 0.1% Tween 20; PFA: paraformaldehyde; RNAi: RNA interference; Sox1: Sry-related high mobility group box 1; SSC: saline sodium citrate; TBST: Tris buffered saline with 0.1% Tween20.

## Competing interests

The authors declare that they have no competing interests.

## Authors’ contributions

TK conceived and designed research, acquired data, analyzed and interpreted data and results, performed statistical analysis, and drafted the manuscript. PT provided study materials, contributed to analysis and interpretation of data, and was involved in drafting the manuscript and providing critical review. MD provided study materials, contributed to analysis and interpretation of data, and was involved in drafting the manuscript and providing critical review. WKL contributed to analysis and interpretation of data, and was involved in drafting the manuscript and providing critical review. SK conceived and designed research, contributed to analysis and interpretation of data, and was involved in drafting the manuscript and providing critical review. ML conceived and designed research, contributed to analysis and interpretation of data, and was involved in drafting the manuscript and providing critical review. All authors read and approved the final manuscript.

## Authors’ information

Simon A Koblar and Martin D Lewis are co-senior authors.

## Supplementary Material

Additional file 1 **Primer sequences and PCR conditions used for each primer set. **^a^These primers were designed for mouse transcripts and therefore contained some nucleotide mismatches to human cDNA sequences (underlined); however, they successfully generated RT-PCR products of the expected size (Npas4 primers - 348 bp; β actin primers - 511 bp) when used in reactions containing cDNA derived from human samples. Click here for file

Additional file 2 **Verification of anti-Npas4 antibody specificity.** The anti-Npas4 antibody was able to detect a protein of approximately 100 kDa in HEK 293 T cells transfected with a recombinant Npas4 expression construct (rNpas4) while no signal was observed in untransfected HEK 293 T cells (n = 3). The expression vector contained the cDNA sequence coding for the mouse Npas4 protein in which the stop codon had been removed and replaced with two copies of the sequence coding for a Myc epitope placed in tandem. The Myc sequences were positioned in frame and were followed by a stop codon such that translation of the resulting mRNA would yield the mouse Npas4 protein fused to two C-terminal Myc tags. An antibody to the reference protein β-actin was used as a loading control. Click here for file

Additional file 3 **Temporal expression of GFP during N2B27 differentiation of the 46C transgenic mESC line.** GFP expression is under the control of the endogenous Sox1 promoter as visualized by fluorescence microscopy (n ≥3). Scale bars = 100 μm. Click here for file

Additional file 4 **Assessment of the pluripotency status of transduced mESC lines.** (A) Stable integration of shRNA constructs into the mESC genome was confirmed by genomic PCR using primers specific to the integration fragment. After 10 passages or more, genomic DNA was isolated from mESC lines that were virally transduced with shRNA expression constructs and also from the untransduced 46C parental mESC line. Primers to the β-actin gene were used as a positive control for genomic DNA isolation. The negative control reaction (−) contained water in place of template genomic DNA. (B) mESC lines were assessed for expression of the pluripotency genes *Pou5f1* and *Nanog* using RT-PCR (n = 3). (C) Percentage of colonies expressing alkaline phosphatase in undifferentiated mESC lines as determined by an alkaline phosphatase detection assay. Means and standard deviations of three independent experiments are shown (n = 3). No statistically significant difference was observed between mESC lines. *P* = 0.5078 (one-way ANOVA). (D) Representative images of colonies from each mESC line after alkaline phosphatase detection assay (Fast Red Violet staining). Scale bar = 100 μm. (E) Comparison of the morphology of undifferentiated mESC lines. Scale bar = 100 μm. (F) Comparison of growth rate between mESC lines. Means and standard deviations of four independent experiments are shown (n = 4). No statistically significant difference was observed between slopes using linear regression. *P* = 0.115. Click here for file

Additional file 5 **Analysis of Nestin expression in various mESC lines after four days of neural differentiation in N2B27 medium.** (A) Immunocytochemical analysis of Nestin expression (red) during neural differentiation. Representative images of cultures after four days of differentiation are shown for each mESC line. Cells were counterstained with DAPI to visualize nuclei (blue). Scale bar = 100 μm. (B) Quantification of Nestin expression in Npas4 knockdown and control mESC lines after four days of differentiation. The number of Nestin-expressing cells (Nestin^+^, DAPI^+^) was counted manually by a blinded researcher and expressed as a percentage of total cells (DAPI^+^). Means and standard deviations of four independent experiments are shown (n = 4). No statistically significant difference was observed between the Empty vector control and the Npas4 KD1 mESC line (*P* = 0.4203, unpaired t test) or the Npas4 KD2 mESC line (*P* = 0.1614, unpaired t test). Click here for file
