# Supplementary material for: A reduction in Npas4 expression results in delayed neural differentiation of mouse embryonic stem cells
Source: Stem Cell Res Ther. 2014 May 8;5(3):64. doi: 10.1186/scrt453 (PMC4076635; doi:10.1186/scrt453)
Supplement: Additional file 1 — Primer sequences and PCR conditions used for each primer set. aThese primers were designed for mouse transcripts and therefore contained some nucleotide mismatches to human cDNA sequences (underlined); however, they successfully generated RT-PCR products of the expected size (Npas4 primers - 348 bp; β actin primers - 511 bp) when used in reactions containing cDNA derived from human samples. [file scrt453-S1.doc]

| **Primer set**  **(species)** | **Application** | **Primer sequences (5’-3’)** | **Final concentration** | **Annealing temperature** | **DNA polymerase** | **Cycles** | **Amplicon size** | | | **Reference** |
| --- | --- | --- | --- | --- | --- | --- | --- | --- | --- | --- |
|  |  |  |  |  |  |  | **cDNA** | | **gDNA** |  |
| Npas4  (mouse) | RT-PCR | TCATGAGTCTTGCCTGCATC^a^ | 500nM | 58°C | Phusion™ Flash | 35 | 347bp | | 1,385bp | - |
|  |  | GAGGGACTTGGAGGTGTTGA^a^ |  |  |  |  |  |  |  |  |
|  | qRT-PCR | AGCATTCCAGGCTCATCTGAA | 0.3pM | 60°C | AmpliTaq Gold® | 40 | 82bp | | 82bp | - |
|  |  | GGCGAAGTAAGTCTTGGTAGGATT |  |  |  |  |  |  |  |  |
|  | ISH probe PCR | TATGAGAAGTTGCCCCCAAG | 60nM | 58°C | Taq  (1mM MgCl_2_) | 35 | 991bp | | 1,649bp | - |
|  |  | CGGTGAGGAAGTGAGACTCC |  |  |  |  |  |  |  |  |
| β-actin  (mouse) | RT-PCR  gDNA PCR | TGTGATGGTGGGAATGGGTCAG^a^ | 600nM | 54°C | Taq  (2.5mM MgCl_2_, 5% DMSO) | 35 | 511bp | | 965bp | - |
|  |  | TTTGATGTCACGCACGATTTCC^a^ |  |  |  |  |  |  |  |  |
|  | qRT-PCR | ACGGCCAGGTCATCACTATTG | 0.3pM | 60°C | AmpliTaq Gold® | 40 | 72bp | | 72bp | - |
|  |  | CCAAGAAGGAAGGCTGGAAAA |  |  |  |  |  |  |  |  |
| Pou5f1  (mouse) | RT-PCR | CCCAGGCCGACGTGG | 400nM | 61°C | Taq  (1.5mM MgCl_2_) | 37 | 65bp | | 250bp | - |
|  |  | GATGGTGGTCTGGCTGAACAC |  |  |  |  |  |  |  |  |
| Nanog  (mouse) | RT-PCR | CAGAAAAACCAGTGGTTGAAGACTAG | 400nM | 61°C | Taq  (1.5mM MgCl_2_) | 37 | 81bp | | 507bp |  |
|  |  | GCAATGGATGCTGGGATACTC |  |  |  |  |  |  |  |  |
| Sox1  (mouse) | RT-PCR | GCCCAGGAAAACCCCAAGATG | 300nM | 54°C | Platinum® Pfx DNA polymerase | 34 | 362bp | 362bp | | - |
|  |  | CCGTTAGCCCAGCCGTTGAC |  |  |  |  |  |  |  |  |
| Nestin  (mouse) | RT-PCR | GCTCAGTGCCGAGCTTGGGG | 300nM | 54°C | Platinum® Pfx DNA polymerase | 24 | 701bp | 1,704bp | | - |
|  |  | CCAGGGCTTCCACAGCCAG |  |  |  |  |  |  |  |  |
| NF-M  (mouse) | RT-PCR | CGCCACAACCACGACCTCA | 300nM | 54°C | Platinum® Pfx DNA polymerase | 26 | 427bp | 2,657bp | |  |
|  |  | CGGCCTCTTCCTTCTCCTCTTT |  |  |  |  |  |  |  |  |
| POU5F1  (human) | RT-PCR | AGTGAGAGGCAACCTGGAGA | 400nM | 55°C | Taq  (1mM MgCl_2_) | 36 | 273bp | 535bp | |  |
|  |  | GTGAAGTGAGGGCTCCCATA |  |  |  |  |  |  |  |  |
| PAX6  (human) | RT-PCR | AATAACCTGCCTATGCAACCC | 200nM | 58°C | Taq  (1mM MgCl_2_) | 28 | 207bp | 717bp | |  |
|  |  | AACTTGAACTGGAACTGACACAC |  |  |  |  |  |  |  |  |
| PAX7  (human) | RT-PCR | AAGATTCTTTGCCGCTACCA | 400nM | 58°C | Platinum® PCR SuperMix HiFi | 35 | 192bp | 1,194bp | |  |
|  |  | CACAGTGCTTCGGTCACAGT |  |  |  |  |  |  |  |  |
| PAX3  (human) | RT-PCR | GCTGTGCCCAGGATGATGCGG | 400nM | 62°C | Platinum® PCR SuperMix HiFi | 35 | 113bp | 1,430 | | - |
|  |  | ACACCGCCGAGCTGGTTGAC |  |  |  |  |  |  |  |  |
| pUC/M13 | ISH probe PCR | GTTTTCCCAGTCACGAC | 400nM | 58°C | Taq  (1mM MgCl_2_) | 35 | ~1,100bp | | | - |
|  |  | CAGGAAACAGCTATGAC |  |  |  |  |  |  |  |  |
| gDNA construct | gDNA PCR | GAGAGAGATGGGTGCGAGAG | 400nM | 58°C | Taq  (0.5mM MgCl_2_) | 25 | 542bp | | | - |
|  |  | CCAAGAACCCAAGGAACAAA |  |  |  |  |  |  |  |  |

**References**

1 Lin T, Chao C, Saito S, et al. p53 induces differentiation of mouse embryonic stem cells by suppressing Nanog expression. Nat Cell Biol 2005; 7:165-171

2 Menzies FM, Grierson AJ, Cookson MR, et al. Selective loss of neurofilament expression in Cu/Zn superoxide dismutase (SOD1) linked amyotrophic lateral sclerosis. Journal of neurochemistry 2002; 82:1118-1128

3 Itsykson P, Ilouz N, Turetsky T, et al. Derivation of neural precursors from human embryonic stem cells in the presence of noggin. Mol Cell Neurosci 2005; 30:24-36

4 Koch P, Opitz T, Steinbeck JA, et al. A rosette-type, self-renewing human ES cell-derived neural stem cell with potential for in vitro instruction and synaptic integration. Proceedings of the National Academy of Sciences of the United States of America 2009; 106:3225-3230
