# Supplementary figures and images for: A reduction in Npas4 expression results in delayed neural differentiation of mouse embryonic stem cells
Source: Stem Cell Res Ther. 2014 May 8;5(3):64. doi: 10.1186/scrt453 (PMC4076635; doi:10.1186/scrt453)

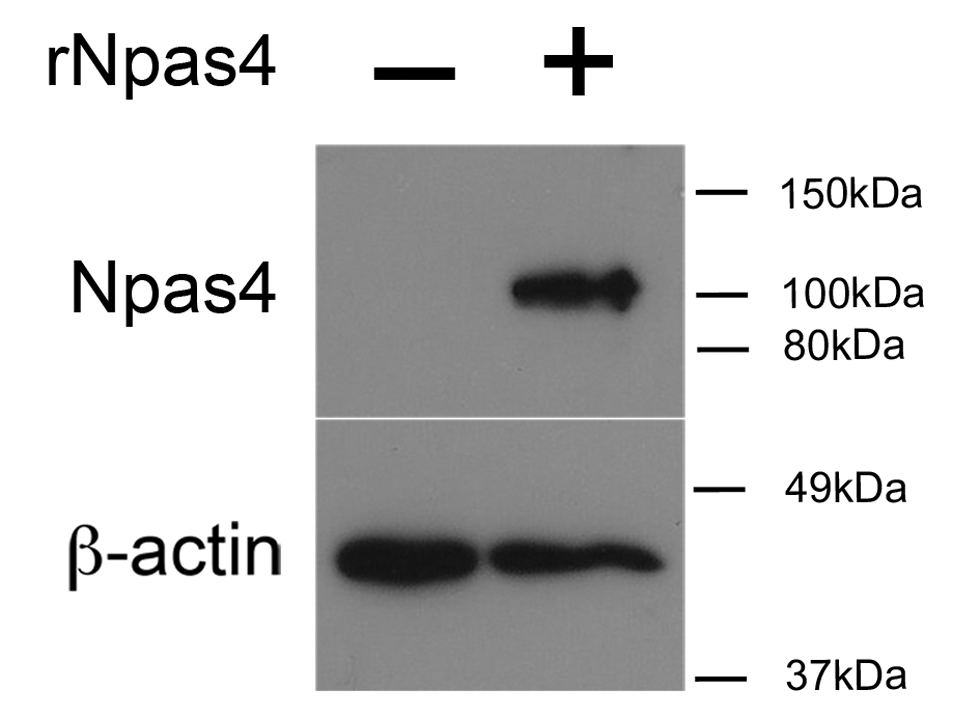

Supplement: Additional file 2 — Verification of anti-Npas4 antibody specificity. The anti-Npas4 antibody was able to detect a protein of approximately 100 kDa in HEK 293 T cells transfected with a recombinant Npas4 expression construct (rNpas4) while no signal was observed in untransfected HEK 293 T cells (n = 3). The expression vector contained the cDNA sequence coding for the mouse Npas4 protein in which the stop codon had been removed and replaced with two copies of the sequence coding for a Myc epitope placed in tandem. The Myc sequences were positioned in frame and were followed by a stop codon such that translation of the resulting mRNA would yield the mouse Npas4 protein fused to two C-terminal Myc tags. An antibody to the reference protein β-actin was used as a loading control. [file scrt453-S2.tiff]

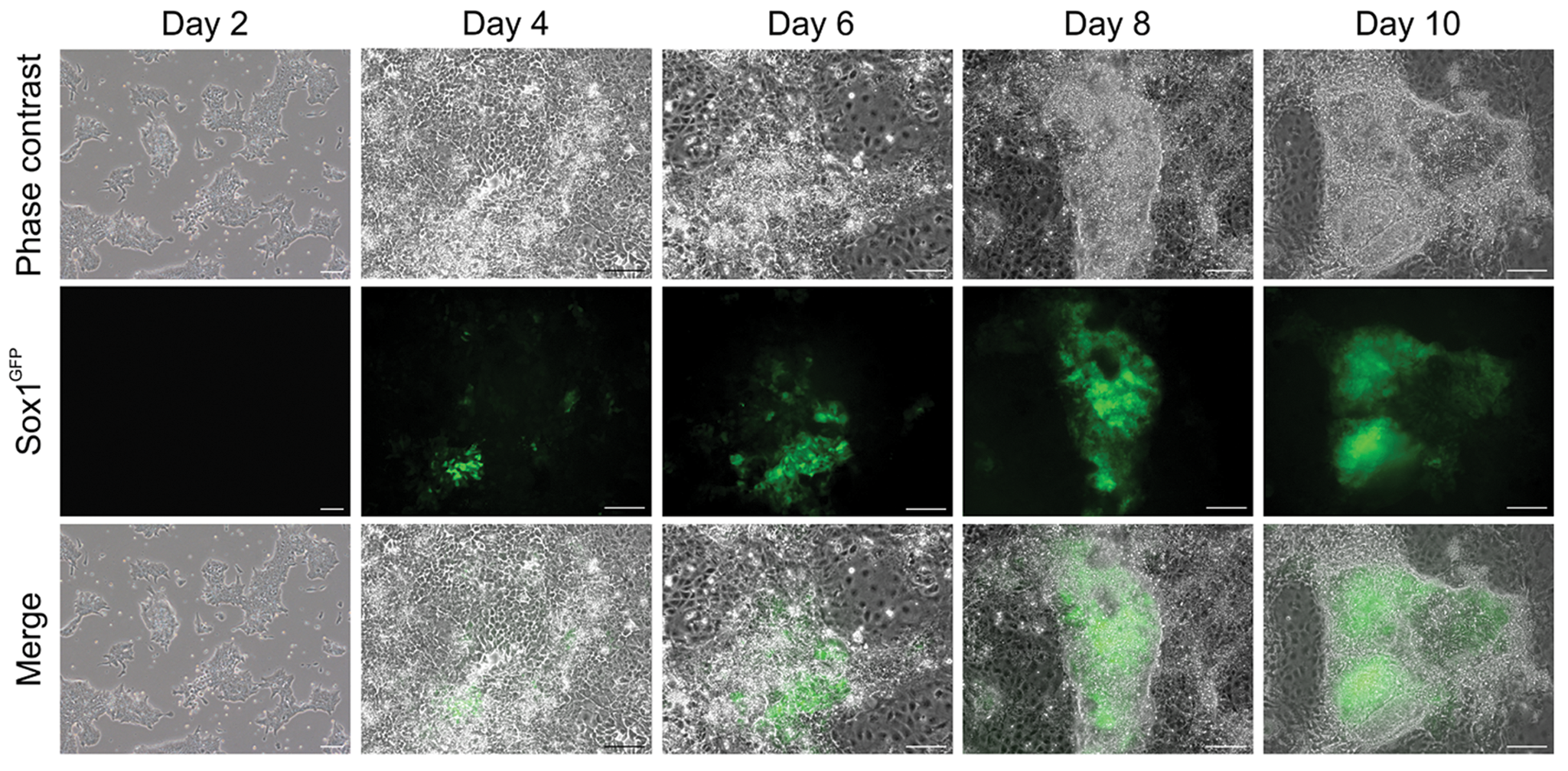

Supplement: Additional file 3 — Temporal expression of GFP during N2B27 differentiation of the 46C transgenic mESC line. GFP expression is under the control of the endogenous Sox1 promoter as visualized by fluorescence microscopy (n ≥3). Scale bars = 100 μm. [file scrt453-S3.tiff]

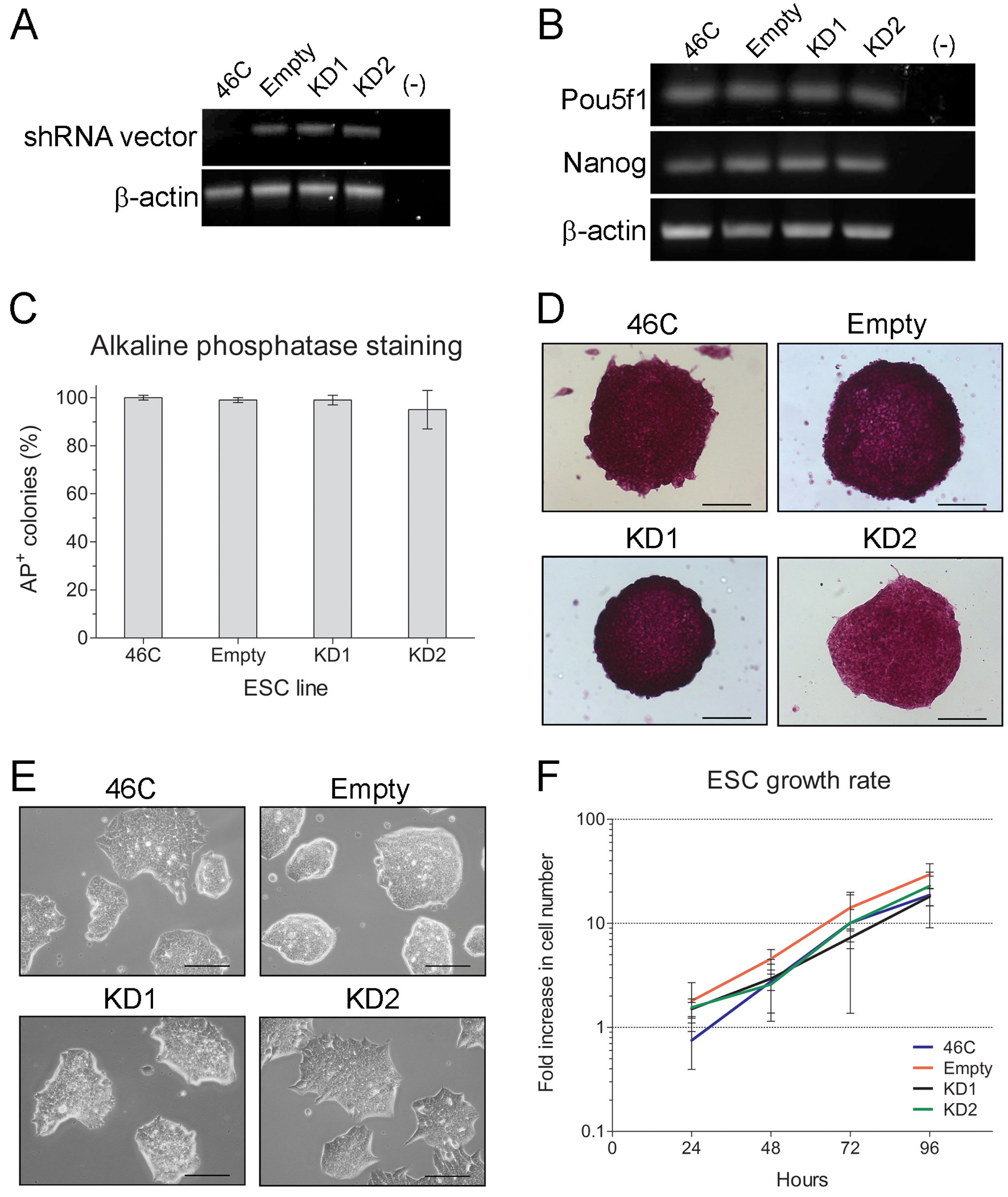

Supplement: Additional file 4 — Assessment of the pluripotency status of transduced mESC lines. (A) Stable integration of shRNA constructs into the mESC genome was confirmed by genomic PCR using primers specific to the integration fragment. After 10 passages or more, genomic DNA was isolated from mESC lines that were virally transduced with shRNA expression constructs and also from the untransduced 46C parental mESC line. Primers to the β-actin gene were used as a positive control for genomic DNA isolation. The negative control reaction (−) contained water in place of template genomic DNA. (B) mESC lines were assessed for expression of the pluripotency genes Pou5f1 and Nanog using RT-PCR (n = 3). (C) Percentage of colonies expressing alkaline phosphatase in undifferentiated mESC lines as determined by an alkaline phosphatase detection assay. Means and standard deviations of three independent experiments are shown (n = 3). No statistically significant difference was observed between mESC lines. P = 0.5078 (one-way ANOVA). (D) Representative images of colonies from each mESC line after alkaline phosphatase detection assay (Fast Red Violet staining). Scale bar = 100 μm. (E) Comparison of the morphology of undifferentiated mESC lines. Scale bar = 100 μm. (F) Comparison of growth rate between mESC lines. Means and standard deviations of four independent experiments are shown (n = 4). No statistically significant difference was observed between slopes using linear regression. P = 0.115. [file scrt453-S4.tiff]

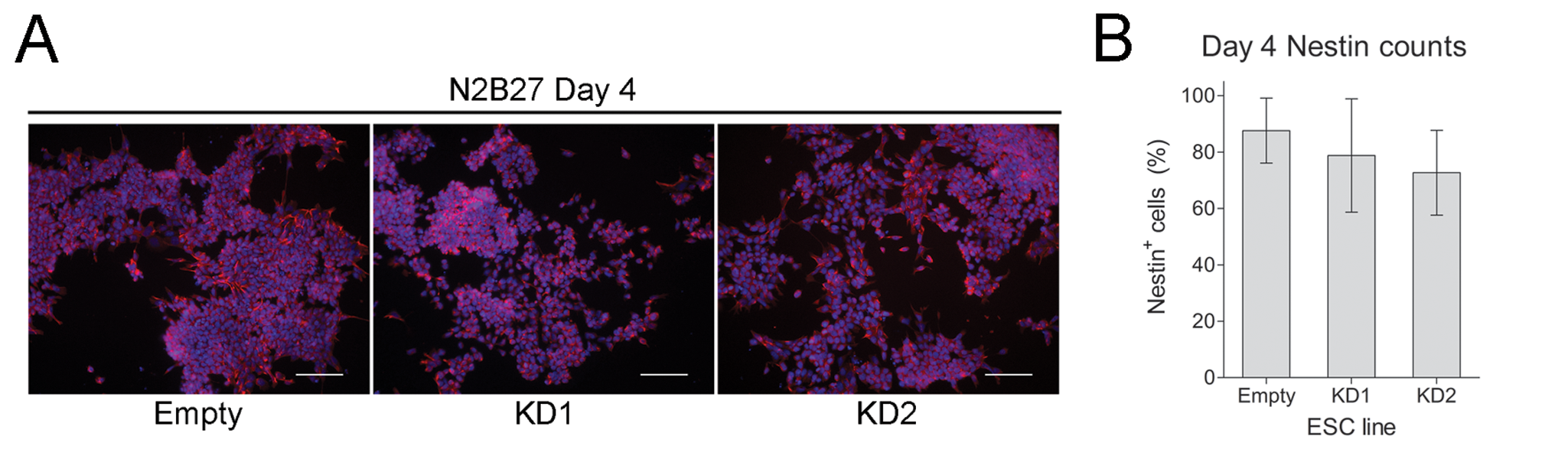

Supplement: Additional file 5 — Analysis of Nestin expression in various mESC lines after four days of neural differentiation in N2B27 medium. (A) Immunocytochemical analysis of Nestin expression (red) during neural differentiation. Representative images of cultures after four days of differentiation are shown for each mESC line. Cells were counterstained with DAPI to visualize nuclei (blue). Scale bar = 100 μm. (B) Quantification of Nestin expression in Npas4 knockdown and control mESC lines after four days of differentiation. The number of Nestin-expressing cells (Nestin+, DAPI+) was counted manually by a blinded researcher and expressed as a percentage of total cells (DAPI+). Means and standard deviations of four independent experiments are shown (n = 4). No statistically significant difference was observed between the Empty vector control and the Npas4 KD1 mESC line (P = 0.4203, unpaired t test) or the Npas4 KD2 mESC line (P = 0.1614, unpaired t test). [file scrt453-S5.tiff]
